# Mitochondrial dysfunction: A promising therapeutic target for liver diseases

**DOI:** 10.1016/j.gendis.2023.101115

**Published:** 2023-09-17

**Authors:** Ping Chen, Lichao Yao, Mengqin Yuan, Zheng Wang, Qiuling Zhang, Yingan Jiang, Lanjuan Li

**Affiliations:** a Department of Infectious Diseases, Renmin Hospital of Wuhan University, Wuhan, Hubei 430060, China; b State Key Laboratory for Diagnosis and Treatment of Infectious Diseases, National Clinical Research Center for Infectious Diseases, Collaborative Innovation Center for Diagnosis and Treatment of Infectious Diseases, The First Affiliated Hospital, College of Medicine, Zhejiang University, Hangzhou, Zhejiang 310003, China

**Keywords:** Chronic diseases, Liver damage, Mesenchymal stem cell, Mitochondrialdysfunction, Oxidative stress

## Abstract

The liver is an important metabolic and detoxification organ and hence demands a large amount of energy, which is mainly produced by the mitochondria. Liver tissues of patients with alcohol-related or non-alcohol-related liver diseases contain ultrastructural mitochondrial lesions, mitochondrial DNA damage, disturbed mitochondrial dynamics, and compromised ATP production. Overproduction of mitochondrial reactive oxygen species induces oxidative damage to mitochondrial proteins and mitochondrial DNA, decreases mitochondrial membrane potential, triggers hepatocyte inflammation, and promotes programmed cell death, all of which impair liver function. Mitochondrial DNA may be a potential novel non-invasive biomarker of the risk of progression to liver cirrhosis and hepatocellular carcinoma in patients infected with the hepatitis B virus. We herein present a review of the mechanisms of mitochondrial dysfunction in the development of acute liver injury and chronic liver diseases, such as hepatocellular carcinoma, viral hepatitis, drug-induced liver injury, alcoholic liver disease, and non-alcoholic fatty liver disease. This review also discusses mitochondrion-centric therapies for treating liver diseases.

## Introduction

Hepatotoxins (drugs, alcohol consumption, viral or bacterial infection, and lipid deposition) or autoimmune response can induce acute liver injury and chronic liver diseases such as viral hepatitis, drug-induced liver injury, autoimmune hepatitis, alcoholic liver disease, and non-alcoholic fatty liver disease (NAFLD).^1^ Hepatic fibrosis is a common complication of almost all types of hepatopathies, and if left untreated, liver fibrosis may eventually progress to cirrhosis, liver failure, and hepatocellular carcinoma (HCC).^2^, ^3^, ^4^ Fibrosis is a dynamic process that can be prevented or reverted by eliminating pathogenic factors or carrying out appropriate therapeutic interventions, such as with antiviral drugs that delay the progression of virus-associated hepatic fibrosis.^5^, ^6^, ^7^, ^8^ Despite these measures, the mortality rate associated with liver diseases has increased from 3% in 2010 to 3.5% in 2019 among all deaths worldwide, thus imposing a huge economic burden globally.^9^ Therefore, elucidating the molecular mechanisms of liver injury and developing new potential therapeutic targets is crucial.

Mitochondria serve as the “power station” of eukaryotic cells and play an important role in metabolizing lipids and saccharides to produce energy in the form of ATP. They also participate in many vital cellular activities, including the urea cycle, iron metabolism, calcium storage homeostasis, cell proliferation, and signal transduction.^10^ Additionally, they control inflammation and the development of related diseases by regulating innate immune responses.^11^ Disruption of these mitochondrial processes may serve as a driving factor for the onset and progression of liver diseases. Furthermore, mitochondria play a role in maintaining the cellular redox state by balancing reactive oxygen species (ROS) production and elimination by the antioxidant defense system. Oxidative stress occurs when impaired mitochondria are unable to scavenge the over-produced ROS, which is considered one of the causative factors for hepatocyte death and liver injury.^12^ Reduction of oxidative stress can inhibit the development of liver fibrosis.^13^ Furthermore, accumulating evidence suggests that agents targeting different types of mitochondrial dysfunction can improve impaired mitochondrial function. This review mainly focuses on the mechanisms underlying mitochondrial dysfunction in liver damage as well as discusses the application of mitochondrion-based therapies in treating liver diseases.

## Mitochondria and its characteristics

### ATP production

Mitochondria are double membrane-bound organelles that possess their genome (mitochondrial DNA, mtDNA), which encodes core protein subunits of the electron transport chain complexes I–V (COX I–V) and ATP synthesis.^14^ Approximately 90% of the generated ATP is produced through the OXPHOS. Briefly, in the mitochondrial matrix, the energy substrates enter into the tricarboxylic acid cycle and generate electron carriers (nicotinamide adenine dinucleotide and flavin adenine dinucleotide) and the electron carriers move through the electron transport chain and stimulate the protons to pump out from the matrix to the intermembrane space, thereby forming an electrochemical gradient termed as the “mitochondrial transmembrane potential” (Δψm).^15^^,^^16^ The COX V converts the energy from proton movement, thus phosphorylating ADP to ATP. Finally, COX IV reaches the final step in the mitochondrial respiratory chain, wherein it accepts electrons from reduced *Cyt c* molecules and transfers them to oxygen and protons, producing water molecules.^17^

### Mitochondial ROS

ROS, which are byproducts of OXPHOS, include not only superoxide anion radical (O2^•−^), hydrogen peroxide (H_2_O_2_), and hydroxyl radical (HO^•^) but also diverse peroxides, such as nucleic acids, lipids, and protein peroxides.^18^ Accumulated ROS is counteracted by antioxidant defense systems comprising enzymatic scavengers such as superoxide dismutase (SOD), catalase, glutathione peroxidase (GPx), non-enzymatic metabolites, ascorbic acid, and glutathione (GSH).^19^ In the mitochondrion, the dismutase activity of SOD enhances the conversion of superoxide anion to H_2_O_2_ and oxygen, after which the H_2_O_2_ decomposes into water, a reaction that is catalyzed by catalase and GPx-1 and requires GSH for enzyme activity. Elevated ROS induces the opening of mitochondrial permeability transition pores (mPTP).^20^ ROS release associated with brief reversible mPTP opening constitutes an adaptive housekeeping function by timely release from mitochondria of accumulated potentially toxic levels of ROS.^21^ However, excessive amounts of ROS cause mPTP opening for a longer time and ΔΨm dissipation; consequently, large amounts of water and ions enter into the mitochondrial matrix, leading to mitochondrial swelling and rupture of the mitochondrial outer membrane, followed by a “burst phase” of ROS production, resulting in oxidative damage to mtDNA, proteins, and lipids.^22^, ^23^, ^24^ Therefore, mitochondria are major producers and targets in terms of ROS.

### Mitochondrial quality control

Mitochondria are continuously undergoing fusion and division to maintain the normal morphology and functional state.^25^ The division process is primarily regulated by Drp1, while the two mitofusins (MFN1 and MFN2) and OPA1 are responsible for the fusion of the mitochondrial inner membrane and mitochondrial outer membrane, respectively^26^ (Fig. 2A). Mitochondria homeostasis is regulated by two processes that have contrary functions: mitochondrial autophagy (mitophagy, selectively eliminates defective mitochondria through fusion with lysosomes) and mitochondrial biogenesis (generates new mitochondria from the existing ones).^27^ PGC-1α is recruited to the chromatin and serves as a pleiotropic regulator of multiple pathways by interacting with nuclear receptors or activating transcriptional factors, thereby promoting mitochondrial biogenesis and metabolic activities.^28^^,^^29^ All these processes form a dynamic network that performs “quality control” of the organelles and restores homeostasis during energy deprivation or after a mitochondrial insult.

**Figure 1:**
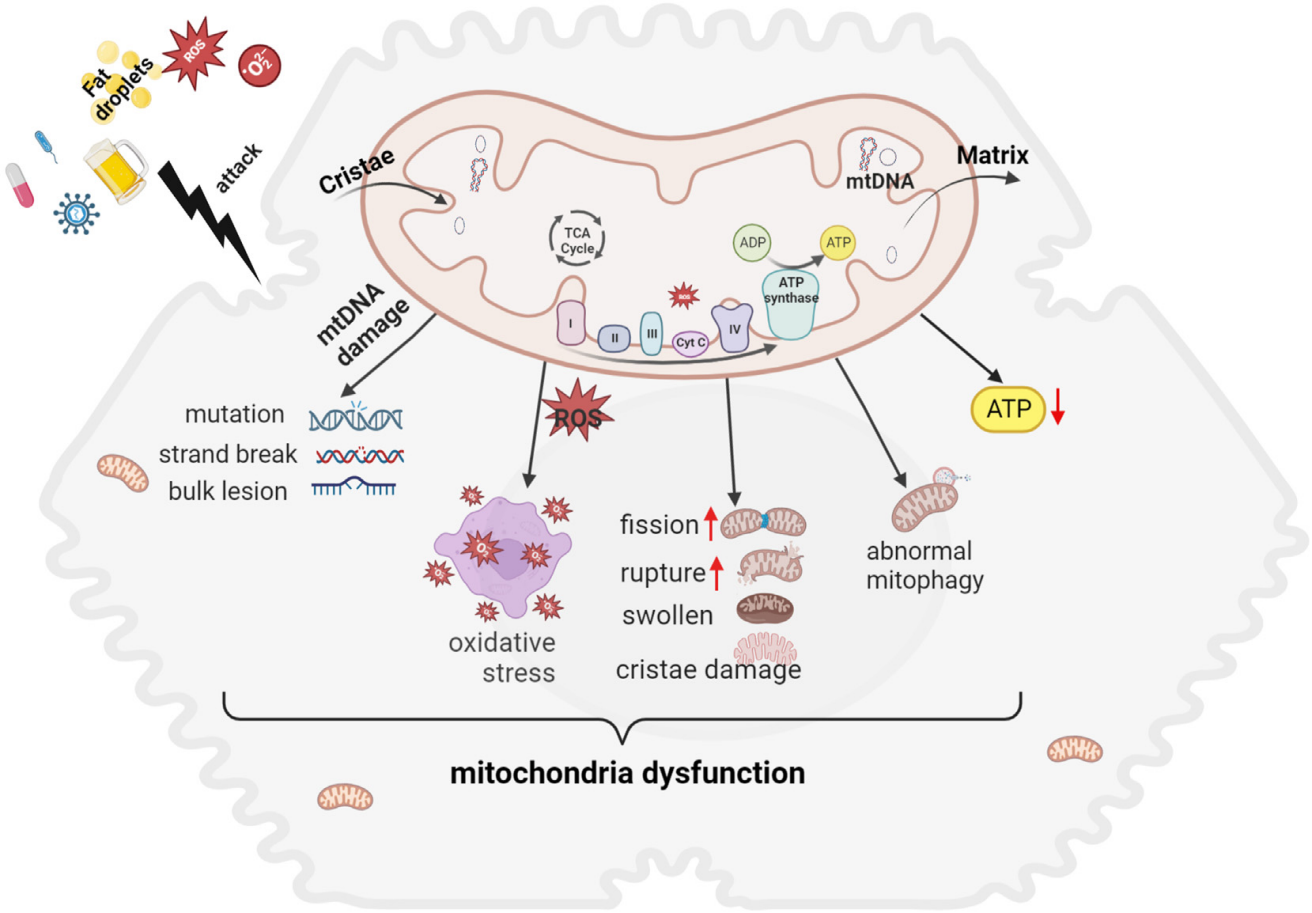
Different types of mitochondrial dysfunction.

**Figure 2:**
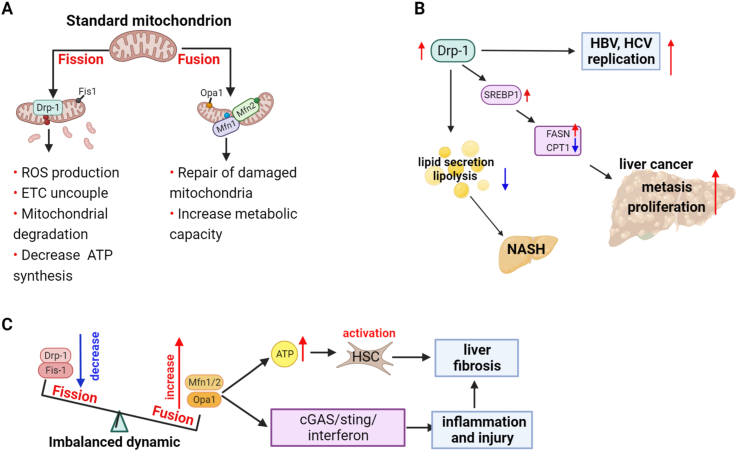
Mitochondrial dynamics and liver diseases. **(A)** Mitochondrial dynamics and cellular activities. **(B, C)** Mechanisms of imbalanced mitochondrial dynamics involved in liver diseases.

Furthermore, ROS can influence mitochondrial dynamics, for example, ROS initiates mitophagy by inducing Parkin to translocate from the cytoplasm to the damaged mitochondria and activating the PINK1/Parkin pathway.^30^ Exogenous addition of ROS promoted mitochondrial fragmentation in fibroblasts.^31^

Mitochondrial dysfunction mainly includes the following aspects: i) impaired mitochondrial “quality control”, wherein there is an imbalance in mitochondrial dynamics, abnormality in mitochondrial biogenesis, and alteration in mitophagy; ii) ROS accumulation; iii) mtDNA damage. Fig. 1 shows the type of mitochondria dysfunctions.

### Liver diseases and mitochondrial dysfunction

The liver is the major metabolic organ in the body and has a strong regenerative and self-repairing ability. Each hepatocyte contains 1000–2000 mitochondria. However, mitochondrial defects or decreased activity of this organelle could reduce ATP synthesis, induce immune dysregulation, stimulate programmed cell death, and delay liver regeneration after an injury.^32^ Mitochondrial dysfunction such as loss of mitochondrial transmembrane potential, decreased activity of mitochondrial respiratory chain complexes, and reduced ATP production contributed to the development of liver fibrosis, cirrhosis, and cancer.^33^, ^34^, ^35^ A recent study showed that markers of hepatic mitochondrial biogenesis, autophagy, fission, and fusion were significantly decreased as NAFLD progressed.^25^ Thus, exploring the mechanism of mitochondrial dysfunction involved in the development of liver diseases is of great significance.

## Impaired quality control of mitochondrial

### Imbalance in mitochondrial dynamics

The mitochondrial morphology is closely related to its functions and is mainly determined by the fission and fusion processes. Both these processes are fine-tuned and influenced by fundamental cellular processes such as calcium homeostasis, ATP generation, and ROS production. Mitochondrial fusion is stimulated by stress and energy demands. Stress-induced fusion allows mitochondria to share components such as electron transport chain complexes that are necessary for ATP generation. Mitochondrial fission usually occurs when new mitochondria are generated and dysfunctional ones are separated. Furthermore, the fission/fusion balance protects cells by modulating mitophagy and mitochondrial biogenesis.^36^

The major pathogenetic mechanism underlying long-term infection with hepatitis B virus (HBV) is the induction of mitochondrial dysfunction, including morphological changes. Ultrastructural analysis of the hepatic cells under a transmission electron microscope revealed that HBV infection resulted in cytoskeletal disruption and mitochondrial morphological abnormalities, including loss of regular tubular or circular shape, disappearance of cristae, and swelling of mitochondria.^37^ Changes in mitochondrial dynamics caused by viral replication (HBV and hepatitis C virus) induce Drp1 expression and promote Parkin translocation, thereby shifting the balance in mitochondrial dynamics towards enhanced division and mitophagy; this event in mitophagy helps suppress innate immune responses in host cells and contributes to viral replication and persistent infection^38^^,^^39^ (Fig. 2B).

The mitochondrial division plays an important role in alcoholic liver disease development. Alcohol consumption promotes mitochondrial fragmentation and enlargement. The presence of megamitochondria in the liver biopsies of alcoholic patients or mice fed with alcohol is considered an important feature of alcoholic liver disease.^40^ Drp-1, an important mitochondrial-shaping protein that serves as a driving factor for organelle fragmentation, is the major mediator driving structural alterations. Liver-specific Drp-1 inactivation exacerbates megamitochondria development and reduces alcohol-induced hepatotoxicity. Palma et al suggested that the emergence of megamitochondria was an adaptive mechanism that may counteract excessive fission and mitophagy and that the use of Drp-1 inhibitor may be a promising therapeutic option for alcoholic liver disease.^41^ However, recent studies have shown that alcohol-fed liver-DRP1 knockout mice had more severe liver injury and fibrosis at an advanced stage. Ma et al confirmed that it is difficult to eliminate megamitochondria through mitophagy because of their size, and accumulation of megamitochondria leads to the accumulation of impaired mtDNA, further activating the cGAS/STING/interferon pathway-mediated inflammation.^42^ The equilibrium between fission/fusion, rather than simply increasing the formation of megamitochondria, is likely essential for maintaining cellular function. Thus, loss of Drp-1 may eventually lead to mitochondrial maladaptation and impaired mitophagy, resulting in dysregulated immune responses that exacerbate liver damage.

The role of mitochondrial dynamics in NAFLD is also noteworthy. High-fat or high-cholesterol diet induces hepatic mitochondrial structural defects and an imbalance in fusion/fission, indicated by loss of cristae, rarefied matrix, low levels of OPA-1 and MFN1/2, and increased expression of Drp-1.^43^ Inhibition of hepatocyte Drp1 early in life mitigated high-fat diet-induced simple hepatic steatosis in mice; the possible underlying mechanism was inhibition of hepatocytic mitochondrial division that led to an increase in proton leak, which not only increased lipid oxidation but also decreased ROS production.^44^ However, Liesa et al recently reported that Drp1 knockdown in older mice exacerbated NASH induced with a high-fat and fructose diet, wherein it worsened intrahepatic lipotoxicity induced by accumulation of non-esterified fatty acids and amplified the mitochondrial integrated stress response in the liver, despite decreasing the total intrahepatic lipid content.^45^ This conflicting result suggests that partial Drp1 inhibition may be effective only in preventing simple steatosis, but it is detrimental to patients who have been with diagnosed NASH. An imbalance in mitochondrial dynamics and accumulation of hepatic lipids and ROS may promote and amplify each other and form a vicious cycle, thus eventually exacerbating liver damage.

### Mitochondrial dynamics and bioenergetic metabolism

Metabolic reprogramming, characterized by up-regulation of glycolysis, is a hallmark of HCC and emerging evidence indicates there was a correlation between mitochondrial dynamics and energy metabolism. Activation of mitochondrial fission significantly promoted the proliferation and metastasis of HCC cells both *in vitro* and *in vivo*, and Drp-1 over-expression promoted *de novo* lipogenesis by enhancing the acetylation of SREBP1 and PGC-1α. The elevated levels of SREBP1 then up-regulated the expression of lipogenic genes FASN and ACC1, while PGC-1α suppressed fatty acid β-oxidation (FAO) by down-regulating CPT1A and acyl-CoA oxidase 1.^46^ Additionally, silencing of OPA1 or MFN1 decreased mitochondrial fusion in HCC cells and tumor organoids of cholangiocarcinoma, resulting in cell apoptosis *in vitro* and tumor growth after tumor cell engraftment in nude mice.^47^ The genome-wide transcriptomic profiling further revealed that inhibition of mitochondrial fusion reduced oxygen consumption and ATP content of cancer cells.

Therefore, mitochondrial dynamics exert a complex role in determining cell fate, and its role depends on the type of disease and the specific context. Maintaining the balance between fission and fusion, rather than simply blocking one or the other, is a promising therapeutic approach for liver diseases (Fig. 2C).

## Abnormal mitochondrial biogenesis

### Mechanism of biogenesis

Mitochondrial biogenesis is necessary for maintaining the turnover, quality, and number of mitochondria, and it is tightly regulated by the members of the PGC-1 family. Mitochondrial transcription factor A (TFAM) is a downstream target of nuclear respiratory factors and drives the replication, transcription, and maintenance of mtDNA.^48^ PGC-1α can activate nuclear respiratory factor 1 and bind to it on the promoter of TFAM, thereby promoting mitochondria synthesis. Additionally, PGC-1α coactivates PPAR to induce the expression of CPT-1 and UCP-2, which are responsible for FAO and uncoupling oxygen consumption from ATP synthesis.^49^^,^^50^ AMPK and Sirt-1 are two metabolic sensors that directly modulate PGC-1α activity through phosphorylation and deacetylation, respectively. Moreover, PGC-1α is required for the induction of ROS-scavenging enzymes, including GPx-1 and SOD2.^51^ Indeed, mitochondrial remodeling and biogenesis are important mechanisms in the adaptation of cellular stress and metabolic changes.^52^

### Mitochondrial biogenesis with liver diseases

Mitochondrial biogenesis is involved in the adaptive response of mice to alcohol-induced metabolic stress. Short-term alcohol feeding up-regulated PGC-1α and mitochondrial respiration, thereby promoting liver alcohol catabolism.^53^ The progression from simple steatosis to definite NASH is strictly associated with decreased mitochondrial function, including reduced biogenesis, FAO, and OXPHO.^25^ Long-term cholestasis suppresses the activation of AMPK/Sirt-1 and fails to activate PGC-1α, further decreasing mitochondrial biogenesis and causing mtDNA depletion and resulting in perpetuated liver damage.^35^ By contrast, liver-specific PGC-1α overexpression observably improves hepatic mitochondrial function, increases complete FAO and tricarboxylic acid cycle flux, and alleviates lipid accumulation both *in vivo* and *in vitro*.^54^ Similarly, chlorogenic acid inhibits hepatic stellate cell (HSC) activation by promoting mitochondrial biogenesis and reduces HMGB1-induced extracellular matrix production in hepatic sinusoidal endothelial cells, reducing lipid accumulation and liver fibrosis in NASH mice.^55^

A previous study suggested that impaired OXPHO was the main cause of insulin resistance, and hepatic insulin resistance is often associated with the accumulation of triglycerides within hepatocytes and NAFLD development.^56^ Intriguingly, mitochondrial biogenesis is also related to energy metabolism. Activation of the PGC-1α/PPARγ/PLIN pathways promotes mitochondrial synthesis and rescues dysfunctional OXPHO and lipidosis in experimental NAFLD.^57^ Resveratrol and ginsenoside enhance hepatic mitochondrial biosynthesis and improve OHPXO functional ability and insulin sensitivity by promoting Sirt-1-mediated deacetylation of PGC-1α.^58^ However, genetic mice models of defective OXPHO, such as muscle-specific deletion of TFAM or hepatocyte-specific knockout of apoptosis-inducing factor have shown an increase in insulin sensitivity, as well as an increase in fatty acid metabolism and glycolysis.^59^^,^^60^ The possible underlying mechanism was that hepatocytes undergo energy metabolic reprogramming to adapt to the reduction of oxidative phosphorylation: increasing anaerobic glycolysis as compensation for ATP production and enhancing the utilization of fatty acids and glucose. Therefore, these findings suggest that moderately increasing or decreasing OXPHO ability may be beneficial for improving impaired metabolic processes. However, whether a long-term decline in OXPHO functionality helps improve NAFLD is inconclusive.

**Figure 3:**
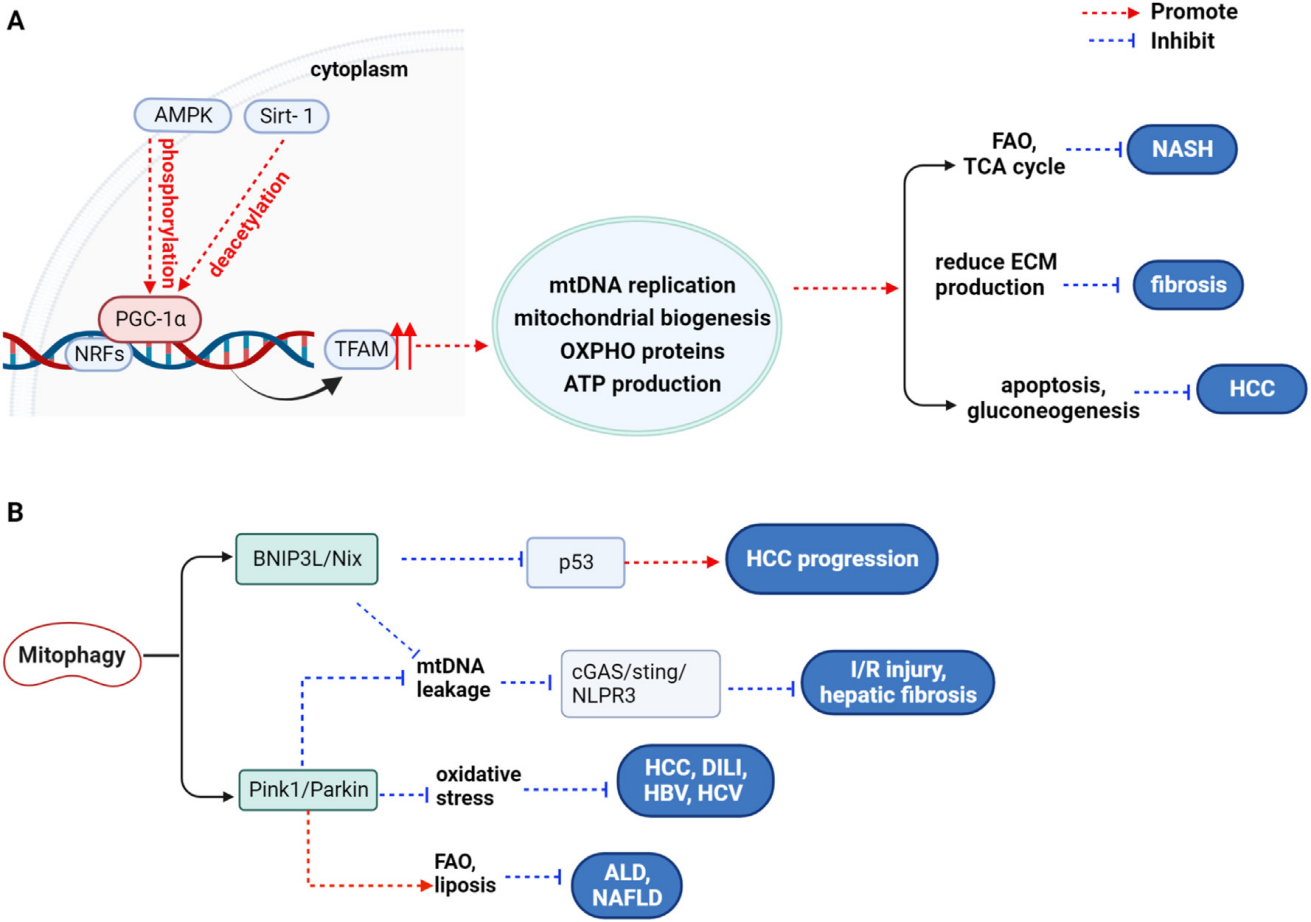
Role of mitochondrial biogenesis and mitophagy in liver diseases.

PGC-1α may act as a tumor suppressor in HCC, and it exerts its function by enhancing mitochondrial biogenesis, mediating gluconeogenesis, and initiating apoptosis pathway.^61^^,^^62^ PGC-1β has a large degree of sequence identity and a similar function to PGC-1α, in sorafenib-resistant HCC cells. The degradation of PGC-1β had increased and was concomitant with a reduction of mitochondrial content and respiratory capacity, resulting in decreased ROS generation in response to sorafenib treatment and sustained drug resistance.^63^ Similarly, oral administration of melatonin attenuates CCl_4_-induced liver fibrosis by improving mitochondrial swelling and preventing the impairment of mitochondria biogenesis and mitophagy^64^ (Fig. 3A).

## Abnormal mitophagy and the development of liver diseases

### Signaling pathways involved in regulating mitophagy

ROS overproduction and damage to mitochondrial proteins and mtDNA damage may lead to irreversible mitochondrial membrane depolarization and decreased Δψm, which are characteristics of mitochondrial damage or dysfunction. Under these conditions, mitophagy reduces the release of mtDNA and production of free radicals by degrading damaged mitochondria and inhibits damage-associated molecular patterns (DAMPs)-induced inflammatory response and secondary liver injury. To date, cellular mitophagy is executed through Pink1/Parkin, BNIP3L/Nix, and FUN14 domain-containing 1 pathways, and Pink1/Parkin has been the most widely studied pathway in mammalians.^65^ Pink-1 is usually undetectable in healthy mitochondria but accumulates in the mitochondrial outer membrane in response to membrane depolarization; it recruits Parkin from the cytosol to the damaged mitochondria and activates Parkin's E3 ubiquitin ligase activity, after which Parkin ubiquitinates mitochondrial outer membrane proteins to recruit autophagy adaptors, including SQSTM1/P62, neighbor of BRCA1 gene (NBR1) and optineurin, which bind to LC3 at autophagosomes, leading to mitochondrial degradation.^66^

### Mitophagy and liver damage

Mitophagy is a critical protective mechanism that prevents cell death and promotes recovery. Long-term alcohol intake leads to the accumulation of dysfunctional mitochondria that exceeds their clearing capacity of mitophagy, after which the impaired mitophagy triggers an inflammatory and fibrotic response by promoting the release of mt-DAMPs.^67^ However, failure of timely clearance of the damaged mitochondria aggravated lipid deposition and peroxidation damage in hepatocytes. Repeated administration of sub-toxic APAP to mice resulted in NAPQI accumulation in mitochondria and induced liver oxidative stress; the JNK pathway activation further amplified the process and led to mPTP opening, decreased Δψm, and finally resulted in hepatic cell death, which significantly aggravated by mitophagy inhibition.^68^ BNIP3 or Parkin-mediated mitophagy inhibition facilitates cytoplasmic leakage of mtDNA in macrophages and activation of the Ccl_4_-induced cGAS/STING/NLRP3 pathway, and consequently enhances the levels of interferon-β, tumor necrosis factor-α, and interleukin-6 in mouse liver, thereby aggravating hepatic injury and fibrosis.^69^^,^^70^ Moreover, deletion of the Parkin gene markedly decreased the levels of mitophagy and FAO and exacerbated alcohol-induced liver injury and steatosis, which may be attributed to severe hepatic mitochondrial swelling, fragmentation, and oxidative stress.^71^ However, Ding et al found that mitophagy still occurred in Parkin knockout mice together with increased hepatocyte proliferation and resistance to APAP-induced liver injury.^72^ We speculated that Parkin knockout might have activated other adaptive autophagy or proliferative pathways. Further studies are needed to clarify and confirm the role of Parkin beyond mitophagy.

Of note, the cross-regulation of mitophagy and mitochondrial synthesis is an important mechanism involved in tissue repair post ischemia-reperfusion (I/R) injury. Impaired autophagy exacerbates oxidative stress and increases necrotic and apoptotic cell death during hepatic I/R injury. Mitochondrial biogenesis and PINK1/Parkin-mediated mitophagy were impaired in mice experiencing partial hepatic I/R, whereas exogenous drugs improved the I/R outcomes by activating the Sirt-1/FOXO3a and PGC-1α/TFAM pathways, increasing mtDNA copy number and recovering mitophagy, and reducing hepatocytes inflammation and death, all of which supported the protective role of mitochondrial quality control process in liver injury.^73^, ^74^, ^75^

### Mitophagy and liver cancer

Unbalanced mitophagy may trigger or accelerate hepatocarcinogenesis, and a negative correlation exists between PINK1 with aggressive progression and poor prognosis of cancer in HCC specimens.^76^ Excessive mitophagy can cause ATP deficiency and intracellular calcium overload, consequently promoting the degradation of filamentous actin and lamellipodium-based migration and invasion of HCC cells.^77^ Wang et al reported that Drp1-mediated fission confers protection to healthy mitochondrial domains from elimination by unchecked PINK1-Parkin activity,^78^ and increased mitochondrial fission enhances mitophagy and cell survival in malignant HCC cells.^79^ HBx is a major cause of HBV-related cancer, and the thyroid protects hepatocytes from HBx-induced ROS damage and carcinogenesis by triggering selective mitophagy through the activation of the PINK1/Parkin pathway.^80^ Over-expression of stomatin-like protein 2 accelerates tumor metastasis through the amplification of mitophagy by interacting with Pink1 and increasing its stability. Blocking mitophagy enhanced the inhibitory effect of lenvatinib on HCC cells.^81^

Cancer stem cells are highly tumorigenic and resistant to chemotherapy. HCC cells treated with mitophagy agonist (CCCP) yield an enhanced population and the cancer stem cells had a better self-renewal ability by suppressing the activity of tumor suppressor p53.^82^ HBx enhanced the cancer stemness of HCC cells by promoting Nix-dependent mitophagy-mediated glycolysis metabolism reprogramming, indicating that Nix might be a potential therapeutic target for HCC.^83^ In conclusion, mitophagy may exert dual roles in liver tumorigenesis and its progression, and the mechanism of mitophagy in HCC varies according to different signaling pathways and cellular contexts (Fig. 3B).

## Oxidative stress

### Definition of oxidative stress

As mentioned previously, functional mitochondria maintain cellular redox homeostasis by balancing the production and elimination of ROS. Oxidative stress is broadly defined as a serious imbalance between ROS generated and the capacity of cells to eliminate them through enzymatic and non-enzymatic antioxidant systems.^84^ Oxidative stress occurs if the antioxidant defense systems are not well matched. The basal level of ROS acts as an important second messenger that is involved in signal transduction, cellular metabolism, cell proliferation, and immune response.^85^ However, excessive ROS activates the cells' apoptotic machinery by inducing mitochondrial permeability transition, DNA damage, and ΔΨm collapse, all of which increase the tendency of mitochondria to release apoptosis-inducing factor and *Cyt c* to the cytoplasm, which eventually induces apoptosis or necrosis through initiating caspase-dependent or -independent cascades.^86^^,^^87^ Additionally, malondialdehyde, which is a product of lipids oxidized by ROS, can reduce the components of electron transport chain and in turn facilitate mitochondrial ROS generation.^88^

### Oxidative stress and liver diseases

**Figure 4:**
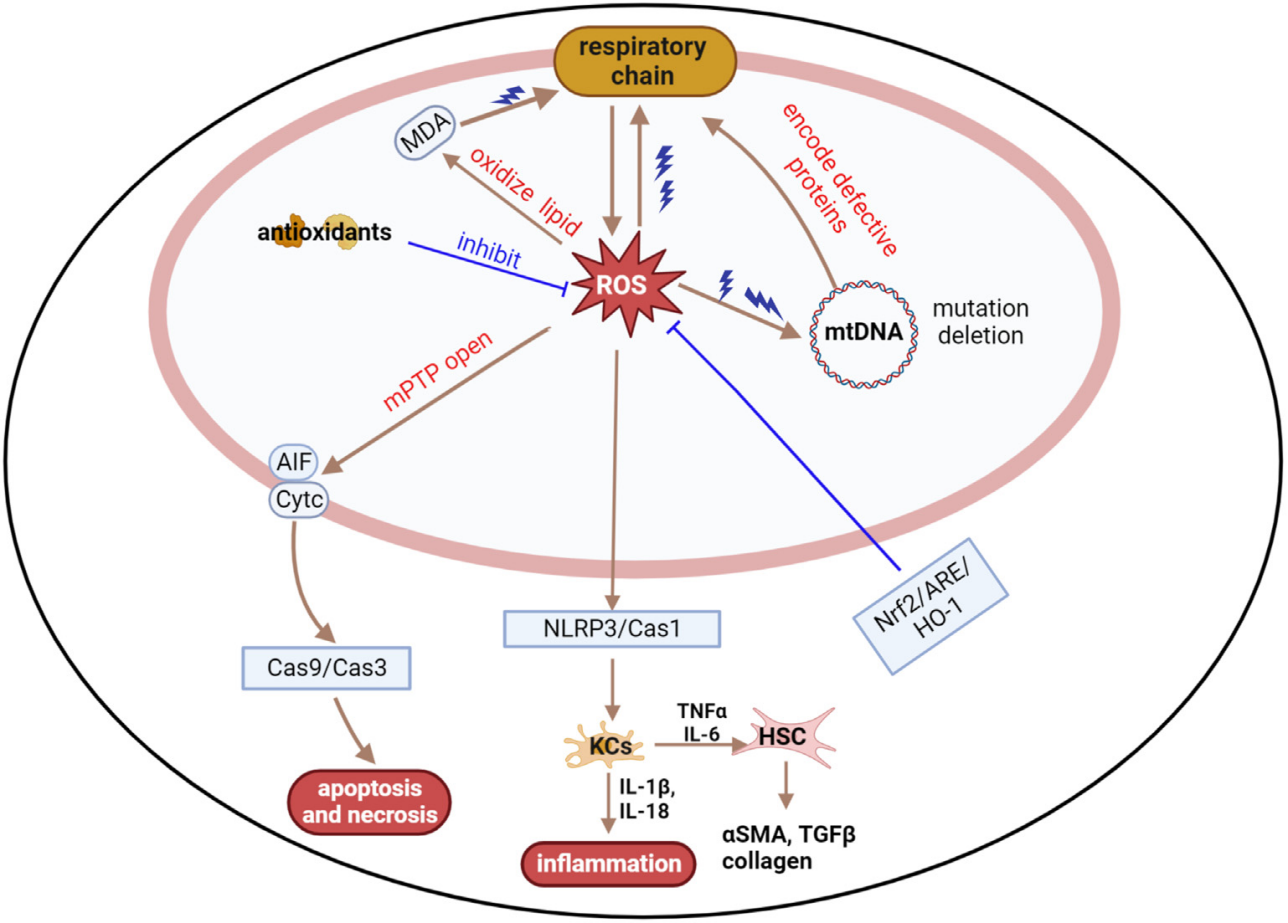
Overview of mitochondrial oxidative stress underlying the pathophysiology of various etiologies of liver diseases.

Oxidative stress is the main factor underlying the pathophysiology of various etiologies of liver diseases (Fig. 4). For instance, drugs such as thiopurine, cyclosporine-A, and paracetamol induce liver damage by directly impairing the activity of mitochondrial respiratory chain, increasing the formation of free radicals and depleting GSH.^89^^,^^90^ CCl_4_ and thioacetamide are well-established hepatotoxic chemicals to induce liver injury, fibrosis, and cancer models. Mechanically, hepatic metabolism of CCl_4_ or thioacetamide releases large amounts of ROS, which in turn triggers Kupffer cells to secret cytokines such as tumor necrosis factor-α and promote hepatic inflammation, as well as tissue necrosis.^91^^,^^92^ Mitochondria in HCC are characterized by the overproduction of ROS, which contributes to tumor progression by inducing genomic instability and modifying gene expression.^93^ Hesperidin acts against CCl_4_-induced hepatocarcinogenesis by increasing the activity of hepatic SOD, GPx, and GST, and suppressing cell proliferation and collagen deposition, and these effects are mediated by activating the nuclear respiratory factor 2/ARE/HO-1 and PPAR-γ pathways.^94^

The role of oxidative stress in liver fibrosis has been highlighted earlier. ROS and apoptotic bodies arising from dying hepatocytes activate HSCs and increase collagen production, which is the pivotal event of liver fibrogenesis. ROS is an activator of the NLRP3 inflammasome, which induces pro-caspase-1 self-cleavage and activation and subsequently promotes the release of the proinflammatory cytokines interleukin-1β and interleukin-18, finally inducing cell death under pathological conditions. Some ROS-producing oxidases, such as NADPH oxidase 4 and the 66-kDa isoform of Shc (p66Shc) promote the activation of liver Kupffer cells and HSCs by activating NLRP3, finally leading to liver inflammatory damage and fibrosis.^95^^,^^96^ Pharmacological induction of HSC apoptosis is a feasible strategy to promote fibrosis regression, Li et al reported that forsythiaside A has anti-fibrosis potential by remolding extracellular matrix and improving oxidation stress to promote apoptosis of HSCs *in vitro*. Forsythiaside A down-regulates the NADPH oxidase 4/ROS signaling pathway to improve oxidation imbalance, decreases collagen-1 and *α*-SMA expression, and increases the ratio of pro-apoptotic Bax to anti-apoptotic Bcl-2.^97^

The ability of ethanol to disrupt the redox balance and induce liver oxidative stress has been established. Long-term ethanol intake significantly increases hepatic cytochrome P4502E1 (CYP2E1) expression and decreases the activity of SOD, catalase, and GPx, both of which promoted ROS generation and make the liver more susceptible to oxidative damage.^91^^,^^98^^,^^99^ Intriguingly, mice fed with antioxidants obviously had decreased alcohol-induced loss of ΔΨm and ROS leakage, increased levels of serum alanine aminotransferase and aspartate aminotransferases, amelioration of hepatocyte apoptosis, and improved liver function.^100^ Sirt1 is a histone deacetylase that regulates lipid metabolism and oxidative stress by deacetylating molecules involved in the tricarboxylic acid cycle, lipogenesis, and FAO. Mice whose liver knockout of Sirt1 and fed with an ethanol-containing diet were more likely to develop hepatic steatosis, inflammation, and fibrosis.^101^

The relationship between oxidative stress and viral hepatitis has attracted the attention of many researchers. ROS-induced liver iron overload acts as a histological characteristic of patients or mice with hepatitis C infection,^102^, ^103^, ^104^ which also shows an intimate correlation with 8-OHdG, a marker of DNA damage.^105^ Serum biomarkers of oxidative stress (H_2_O_2_ and malondialdehyde) are present at increased concentrations in patients infected with HBV or hepatitis C virus, while the antioxidant levels (total sulfhydryl, vitamins C and E, uric acid, *etc*.) are decreased.^106^^,^^107^ The increase in oxidative stress is associated with the HBV genotype, drug resistance mutation, and severity of chronic hepatitis B infection.^108^^,^^109^ Gao et al reported that HBx sensitizes hepatocytes to oxidative stress-induced apoptosis through modulation of the mPTP opening, which affects mitochondrial biogenesis.^110^ HBV-specific CD8 T cells are functionally exhausted in patients with chronic hepatitis B infection, whereas mitochondrion-targeted antioxidants significantly improve the mitochondrial and antiviral functions of CD8 T cells, which suggests the pivotal role of ROS in T-cell exhaustion in chronic hepatitis B infection.^111^

Given that oxidative stress and mitochondrial dysfunction are key factors in the pathogenesis of NAFLD, antioxidants have been used as a therapeutic approach. Nevertheless, the clinical benefits of antioxidant therapy are not as expected.^112^ The possible mechanism is that oxidative stress may be exacerbated when antioxidant defenses are not well matched. NASH is a spectrum of NAFLD characterized by the over-production of superoxide, GSH depletion, and reduced SOD2 activity. Increasing SOD levels in the body appear to have beneficial effects on alleviating oxidative damage. However, Montfort et al demonstrated that SOD2 over-expression or use of SOD mimetics increased H_2_O_2_ levels, and aggravated liver damage and fibrosis in NASH mice, despite reduced superoxide production.^113^ When replenished with GSH, the above phenomenon could be rescued. The logical explanation is that over-production of H_2_O_2_ can deplete GSH and sensitize hepatocytes to oxidative necrosis.

Taken together, these studies suggest that ROS participates in the progression of various liver diseases by inducing hepatocyte damage, apoptosis, and HSC activation. Sometimes the use of superoxide scavengers in combination with GSH supplements may help achieve better efficacy than one of them alone.

### mtDNA damage

Compared with nuclear DNA, mtDNA is vulnerable to ROS attack and prone to mutation owing to the lack of protection from histones and DNA repair systems. Extensive mtDNA damage can exacerbate oxidative stress and destroy the mitochondrial respiratory chain and energy metabolism, thus contributing to the pathogenesis of liver diseases.

### mtDNA mutation and impaired replication

Cytochrome b was a key component of mitochondrial respirasome, Sookoian et al reported that mtDNA mutation at cytochrome b region of liver tissue was more common in patients with advanced NASH and severe obesity, and they had a severe hepatic oxidative injury and impaired metabolic function.^114^

mtDNA mutation and copy number variation are common events in viral hepatitis and HCC. In patients with chronic hepatitis B infection, mtDNA strand breaks and deletion are more serious in patients with cirrhosis than in patients with no/mild-to-moderate fibrosis.^115^ Additionally, the level of mtDNA content in peripheral blood was negatively correlated with HBsAg, HBV viral load, and alanine aminotransferase levels.^116^ Liver tissues from patients with HBV-HCC exhibited numerous mtDNA mutations and a lower copy number than those from healthy control subjects, which contributes to hepatocarcinogenesis and tumor metastasis.^117^^,^^118^ The release of mtDNA into the cytosol induces cytosolic mtDNA stress, consequently promoting the infiltration of CCL2-induced tumor-associated macrophages and HCC progression,^119^ suggesting that mtDNA may be a potential novel non-invasive biomarker to evaluate the risk of progression to cirrhosis and HCC in patients with HBV.

Medici et al observed that the mtDNA copy number in the blood sample of patients with Wilson disease was lower than that in healthy control subjects, and it was inversely correlated with the levels of circulating bilirubin. The authors proposed that direct interaction between mitochondrial copper and mtDNA leads to mtDNA depletion and replication cessation, deterioration of mitochondrial FAO capacity, ATP deficiency, impaired heme synthesis, and hepatic oxidative damage.^120^

### mtDNA leakage

DAMPs can amplify the sterile inflammation induced by dying hepatocytes, trigger innate immune responses, and promote the trans-differentiation of quiet HSCs to fibrotic HSCs, hence increasing the secretion of collagen and other fibrotic factors.^121^ As a major active component of mitochondrial DAMPs (mt-DAMPs), mtDNA can be released from the mitochondria matrix into cytoplasm under pathological conditions. Bax/Bak and voltage-dependent anion channels are two main pathways facilitating mtDNA leakage. They oligomerize at the outer mitochondrial membrane, which increases membrane permeability and allows the release of mtDNA and apoptotic factors.^122^^,^^123^ Cytosolic mtDNA leakage activates TLRs, inflammasomes, and cGAS-STING signaling axis and recruits immune cells to the injury site to trigger inflammatory responses by promoting the production of type I interferon and other immune mediators.^70^^,^^124^, ^125^, ^126^

Drug-induced liver injury is strictly related to mtDNA leakage. Overdose of APAP and alcohol consumption increased serum mtDNA, leading to activation of JNK2/caspase1/TLR9 pathway, enhancement of endoplasmic reticulum stress, and development of hepatocyte damage and liver failure.^11^^,^^127^ Similarly, Meng et al recently reported that mice with specific knockout of hepatic Sam50 promoted Bax mitochondrial recruitment, increased mtDNA leakage, and activated the cGAS-STING axis, which eventually aggravated APAP-induced liver necrosis in mice.^128^ Furthermore, an *in vitro* model of hepatic I/R suggested that mtDNA co-culture with hepatocytes significantly decreased the cell viability in a dose-dependent manner.^129^ By contrast, reducing mtDNA release by enhancing autophagy minimized the adverse effect of hepatic I/R injury.^130^ An et al reported that serum mtDNA is increased in patients with NASH, especially those with severe fibrosis. They also revealed that FVB mice showed resistance to thioacetamide-induced liver fibrosis, whereas injection of hepatocyte-derived mt-DAMPs not only compromised the resistance but also directly activated pro-fibrogenic HSCs.^131^

Thus, scavenging circulating mtDNA might be a potential therapy for hepatic damage and its complications. Depleting mtDNA with ethidium bromide abrogated the activation of the GAS/STING/TBK1/NF-κB pathway in macrophages and alleviated inflammatory liver injury. Furthermore, tetramethylpyrazine prevented liver fibrotic injury by suppressing the release of injured hepatocyte-derived mtDNA, reducing the delivery of mtDNA-containing extracellular vesicles from hepatocytes into HSCs, and improving hepatocyte death and HSC activation caused by mtDNA.^132^

## Therapies and perspectives

### Drug target on mitochondrial dysfunction

Accumulating preclinical evidence has confirmed that mitochondria are involved in the pathogenesis of different liver diseases by regulating various signaling pathways. Currently, therapeutic strategies for mitochondrial dysfunction include supplementing antioxidants (MitoQ, vitamins C/E, and lipoic acid), increasing mitochondrial biogenesis (cilostazol, bezafibrate, epicatechin, and RTA 408), improving the function of electron transport chain (coenzyme Q10, idebenone, vitamin B2, and thiamine), viable mitochondria transplantation, and mesenchymal stroma cell (MSC)-based therapy. Fortunately, some agents have shown satisfactory outcomes in the treatment of liver diseases (Table 1). Idebenone alleviates fatty liver diseases by binding with PPAR-α and regulating lipid metabolism.^133^ The phosphodiesterase inhibitor cilostazol increased the expression of PGC-1α, nuclear respiratory factor 1, and TFAM in hepatocytes, which also up-regulated the expression of COX III and COX IV and increased the content of mtDNA. This agent alleviates I/R-induced hepatic mitochondrial damage, and experimental evidence is available for the clinical application of phosphodiesterase inhibitors in treating I/R injury.^134^ Quercetin protected hepatocytes from ethanol-induced pyroptosis by scavenging mitochondrial ROS and promoting PGC-1*α*-mediated mitochondrial homeostasis in L02 cells.^135^ MitoQ specifically scavenges mitochondrial superoxide and restores immune function in patients with chronic HBV infection.^111^ Evidence from clinical trials has also confirmed that the supplementation of antioxidants helps improve liver function in patients with NASH. Sanyal et al reported that vitamin E supplementation observably reduced the alanine aminotransferase level compared with the placebo, and the reduction was associated with amelioration of steatohepatitis.^136^ At present, resistance to both chemotherapy and immunotherapy remains an obstacle in HCC treatment. Surprisingly, some mitochondrion-targeting drugs have shown good synergistic action when combined with chemotherapeutics. Icaritin provoked immunogenic cell death in HCC cells by enhancing mitophagy and apoptosis. Icaristin together with doxorubicin or lenvatinib participated in the remodeling of the immunosuppressive tumor microenvironment and triggered a robust immune response, which effectively inhibited tumor progression and prolonged survival time in an HCC mice model.^137^ Similarly, dehydrocrenatidine induces cancer cell death by affecting oxidative phosphorylation and inducing ROS accumulation and Δψm disruption.^138^ Sorafenib, when used in combination with dehydrocrenatidine, showed a stronger anti-tumor efficacy without significant side effects.

**Table 1:** Therapeutic intervention for mitochondria in different liver models.

Therapeutics	Models	Main effects	Route of administration	Reference
Cilostazol	Ischemia-reperfusion injury	Increases PGC-1α expression and mtDNA content	Oral	134
Coenzyme Q10	Liver fibrosis	Enhances autophagy	Oral	143
Idebenone	Nonalcoholic steatohepatitis	Regulates lipid metabolism	Oral	133
Quercetin	Alcoholic liver disease, acute liver injury	Scavenges mitochondrial reactive oxygen species; increases the anti-apoptotic and anti-inflammatory potential	Oral	135,144
MitoQ	Hepatitis B virus	Decreases superoxide formation, restores immune function	Oral	111
Vitamin E	Nonalcoholic fatty liver disease	Reduces reactive oxygen species and alanine transaminase	Oral	136
Icaritin, **dehydrocrenatidine**, **dichloroacetate**	Hepatocellular carcinoma	Enhances mitophagy; recovers Δψm; inhibits mTOR complex 1; suppresses tumor growth	Oral	137,138,144
**Mitochondria**	Drug-induced liver injury	Reduces oxidative stress and apoptosis	Spleen injection	140
Mesenchymal stroma cells-extracellular vesicles	Nonalcoholic fatty liver disease, ischemia-reperfusion injury	Increases mitochondrial respiratory chain activity and adenosine triphosphate production	Injection	141,142

### Mitochondrial transfer and MSCs

As a novel mode of intercellular communication, mitochondrial transfer can inhibit oxidative stress and restore bioenergetics, thus promoting the recovery of damaged tissues or organs. The ways by which mitochondrial transfer can be achieved mainly include microinjection, incubation with intact purified mitochondria, tunnel nanotubes or gap junction channel-mediated cell attachment, and direct transfer from donor cells such as MSCs.^139^ Intrasplenic infusion of viable mitochondria reduced mitochondrial oxidative stress, *Cyt c* release, and hepatocyte necrosis, thus successfully improving APAP-induced liver tissue and functional deterioration with superior efficacy to N-acetylcysteine, the clinically approved antidote for APAP-induced liver injury.^140^ MSC-based therapy is an effective method for repairing liver injury and enhancing liver regeneration. Zheng et al reported that MSCs exert an anti-apoptotic effect on liver I/R injury by activating AMPKα and up-regulating PINK1-dependent mitophagy. BMSCs improve diabetes-associated NAFLD by promoting their mitochondrial transfer to the liver, thus enhancing OXPHOS activity, ATP generation, and Δψm.^141^ Extracellular vesicles containing a variety of bioactive mitochondrial components such as mtDNA and OXPHOS-related proteins, and extracellular vesicles isolated from hUC-MSC ameliorate hepatic I/R injury by transferring functional mitochondria to neutrophils and inhibiting the formation of neutrophil extracellular traps in liver tissues.^142^

## Challenges and future directions

In this review, we comprehensively summarize the common types of mitochondrial dysfunction and the underlying mechanisms and describe the mechanism of therapies targeting mitochondria in various liver diseases. Mitochondria are vulnerable organs susceptible to drugs, infections, immune system dysregulation, and other such factors. All these etiologies can induce mitochondrial dysfunction and manifest as mtDNA damage, morphological change, energy deficiency, oxidative stress, and inflammatory responses, thus contributing to liver damage. Therefore, it is imperative to improve the understanding of the process and mechanisms of mitochondrial dysfunction in liver diseases. As a new strategy, mitochondrial transplantation therapy is attracting the attention of many researchers. However, limitations in terms of the sources, immunogenicity, instability, and activity of mitochondria seriously restrict the further development of this strategy. Thus, more research is needed on this topic to prevent and treat chronic liver diseases through mitochondrion-centric pathways in the future.

## Author contributions

LJL and YAJ presented the idea and designed the whole outline of this article. PC and LCY contributed to the manuscript writing. MQY, ZW, and QLZ contributed to figure and table preparation. YAJ and LJL reviewed and supervised the manuscript. All authors read and approved the submitted version.

## Funding

The article was funded by the Anti-aging Research Center of Wuhan University Education Development Foundation, Hubei, China (No. 2002330), and the National Stem Cell Clinical Research Project of China (China Medical Biotechnology Association 2019; No. 007).

## Conflict of interests

The authors declare that they have no competing interests.
